# Application of CRISPR/Cas-based gene-editing for developing better banana

**DOI:** 10.3389/fbioe.2024.1395772

**Published:** 2024-08-16

**Authors:** Leena Tripathi, Valentine O. Ntui, Jaindra N. Tripathi

**Affiliations:** International Institute of Tropical Agriculture (IITA), Nairobi, Kenya

**Keywords:** banana, CRISPR/Cas, gene editing, disease resistance, nutrition enhancement

## Abstract

Banana (*Musa* spp.), including plantain, is one of the major staple food and cash crops grown in over 140 countries in the subtropics and tropics, with around 153 million tons annual global production, feeding about 400 million people. Despite its widespread cultivation and adaptability to diverse environments, banana production faces significant challenges from pathogens and pests that often coexist within agricultural landscapes. Recent advancements in CRISPR/Cas-based gene editing offer transformative solutions to enhance banana resilience and productivity. Researchers at IITA, Kenya, have successfully employed gene editing to confer resistance to diseases such as banana Xanthomonas wilt (BXW) by targeting susceptibility genes and banana streak virus (BSV) by disrupting viral sequences. Other breakthroughs include the development of semi-dwarf plants, and increased β-carotene content. Additionally, non-browning banana have been developed to reduce food waste, with regulatory approval in the Philippines. The future prospects of gene editing in banana looks promising with CRISPR-based gene activation (CRISPRa) and inhibition (CRISPRi) techniques offering potential for improved disease resistance. The Cas-CLOVER system provides a precise alternative to CRISPR/Cas9, demonstrating success in generating gene-edited banana mutants. Integration of precision genetics with traditional breeding, and adopting transgene-free editing strategies, will be pivotal in harnessing the full potential of gene-edited banana. The future of crop gene editing holds exciting prospects for producing banana that thrives across diverse agroecological zones and offers superior nutritional value, ultimately benefiting farmers and consumers. This article highlights the pivotal role of CRISPR/Cas technology in advancing banana resilience, yield and nutritional quality, with significant implications for global food security.

## 1 Introduction

Banana including plantain (*Musa* spp.) plays a pivotal role in global agriculture and food security, provides a reliable source of affordable and nutritious food. With its year-round availability and adaptability to diverse climates in the tropics and subtropics (Jones, 2000), banana emerge as a steadfast source of essential nutrients, particularly potassium, vitamin C, vitamin B6, and dietary fiber (Kumari et al., 2023). It not only provides a swift and convenient energy boost but also assumes a central role as a staple in the diets of millions of people in regions, especially within tropics.

In addition to its nutritional value, the economic significance of banana cannot be overstated. Serving as a critical export commodity for numerous tropical nations, banana contributes substantially to international agricultural trade, with vast plantations spanning over 140 countries and islands, covering over 12 million hectares globally, and 7.5 million hectares in Africa (FAOSTAT, 2021). The undeniable ubiquity and economic impact of banana underscore their pivotal role in sustaining the wellbeing of populations worldwide.

However, despite its importance, banana production faces a multitude of challenges, including biotic and abiotic stresses, declining soil fertility, limited genetic diversity, and insufficient availability of clean planting material, particularly among smallholder farmers. The prevalence and co-existence of various pathogens and pests further exacerbate these challenges, leading to significant yield gaps and threatening the sustainability of banana cultivation in affected regions (Tripathi et al., 2020). In response to these pressing challenges, the application of cutting-edge technologies such as CRISPR/Cas-based gene editing emerges as a promising avenue for revolutionizing banana production. By harnessing the power of gene editing, researchers are developing improved banana varieties with enhanced resistance or tolerance to biotic and abiotic stresses, thereby bolstering productivity and resilience in the face of environmental adversities. This article provides a comprehensive overview of recent advancements and future prospects in the utilization of gene editing technologies for the development of better banana varieties. This article seeks to elucidate the potential of CRISPR/Cas-based gene editing in driving innovation and sustainability within the global banana industry.

## 2 Challenges in banana production

Banana production grapples with an array of biotic and abiotic challenges that pose significant threats to both yield and fruit quality. These challenges are multifaceted and contingent upon factors such as geographical location, climate conditions, and specific agricultural practices. Among the notable biotic constraints, bacterial, fungal, and viral diseases stand out as major threats to banana crops.

Particularly menacing are diseases like Fusarium wilt disease, caused by the *Fusarium oxysporum* f. sp. *cubense* (Foc) fungus, and black Sigatoka, attributable to the *Mycosphaerella fijiensis* fungus. These diseases have the potential to inflict substantial yield losses, with Fusarium wilt, in particular, proving to be one of the deadliest biotic constraints (Ploetz, 2015). Fusarium wilt, also known as Panama disease, damages the plant’s vascular system, causing wilting and, ultimately, death. Compounding the challenge is the soilborne nature of Foc, which can persist in the soil for decades, posing significant challenges for disease management (Hennessy et al., 2005).

Furthermore, the emergence of Foc tropical race 4 (TR4) poses a grave threat to banana production globally. This deadly fungal strain has been identified in various regions worldwide, including key banana-producing countries like Mozambique, Colombia, Israel, Jordan, Turkey, Mayotte, Peru, and Venezuela. The spread of TR4 in Africa is particularly alarming, given the continent’s status as the world’s second-largest banana producer and consumer (Viljoen et al., 2020). With limited control, prevention, and management tools currently available, addressing the spread of TR4 remains a critical challenge for sustaining banana production (Ploetz, 2015). The only viable option is disease-resistant banana varieties that ensures increase productivity with high nutritional value by application of genetic engineering (Dale et al., 2017). These TR4 resistant banana are recently approved for environmental release in Australia (OGTR, 2024).

Second, the most important fungal disease is Black Sigatoka, affecting mainly the lower leaves of banana plants and directly reducing the yield of the crop and poor quality of fruits (Arango Isaza et al., 2016).

In addition to fungal diseases, bacterial infections such as banana Xanthomonas wilt (BXW), caused by *Xanthomonas campestris* pv. *musacearum*, pose significant threats to banana production in Africa. The impact of BXW disease on banana yield losses is particularly severe in the Democratic Republic of Congo (83%), Uganda (71%), and other East African countries like Burundi, Kenya, Rwanda, and Tanzania, where losses range from 39% to 51% (Ainembabazi et al., 2015). Notably, the lack of disease-resistant banana varieties exacerbates the economic repercussions of BXW, with estimated losses ranging from USD 2 to 8 billion over a decade (Abele and Pillay, 2007; Biruma et al., 2007; Tripathi et al., 2009). No cultivated banana varieties have yet to demonstrate resistance except for the wild-type diploid banana *Musa balbisiansa,* which is native to Southeast Asia (Nakato et al., 2019).

Several viruses, including *banana streak virus* (BSV, genus Badnavirus) and *banana bunchy top virus* (BBTV, genus Babuvirus) also affect banana production worldwide because of their effects on yield, quality, and limitations to the international germplasm exchange due to presence of viruses in planting materials, posing a severe threat to food and nutrition security in banana-growing regions (Kumar et al., 2015).

Banana also face pressure from various pests, including aphids, mites, nematodes, and weevils, further complicating pest management in banana cultivation. Plant-parasitic nematodes and weevils pose a significant global threat to banana cultivation, resulting in severe yield losses varying from 40% to 50% (Gold et al., 2001). Various nematodes, such as *Radopholus similis*, *Pratylenchus goodeyi*, *Pratylenchus coffeae*, *Helicotylenchus multicinctus*, and *Meloidogyne* spp., are prevalent either alone or in combination in banana fields (Coyne et al., 2013). Similarly, banana weevils (*Cosmopolites sordidus*) stand out as the most challenging insect pest on a global scale, inflicting severe damage to both roots and pseudostems (Twesigye et al., 2018). The escalating impact of these pests underscores the urgent need for sustainable and accessible pest management strategies.

Moreover, abiotic factors such as soil erosion, nutrient deficiencies, and climatic variability pose additional challenges to banana production. The susceptibility of banana to climatic conditions, coupled with the looming threat of climate change, further exacerbates production challenges, potentially altering traditional growing regions and exposing banana to new risks.

Addressing these multifaceted production constraints requires a holistic approach encompassing improved agricultural practices, disease-resistant varieties, sustainable soil management, and strategies to enhance genetic diversity. By adopting such a comprehensive approach, the banana industry can mitigate risks, enhance productivity, and ensure the long-term sustainability of banana cultivation.

## 3 Genetic diversity in banana germplasm

Edible banana were originated through the natural hybridization of two wild progenitors, Musa acuminata (AA genome) and *Musa balbisiana* (BB genome), boast a rich tapestry of genetic diversity. This diversity is reflected in the multitude of cultivars, which are classified into various genome groups based on their genetic makeup. Among these groups are diploid banana genomes, denoted as AA or AB, and seedless triploid genomes, encompassing AAB, AAA, and ABB variations (Hinge et al., 2022). The global count of banana cultivars ranges from an estimated 300 to 1,200, showcasing the wide array of genetic variations that have emerged through natural processes and human interventions (Ploetz et al., 2007; Thierry, 2020). Diploid seeded banana are about 290 cultivars grown in Southeast Asian countries, and edible seedless triploid banana are about 650 cultivars grown worldwide (Thierry, 2020).

The importance of genetic diversity in banana cannot be overstated, particularly in the context of adapting to environmental stresses. A diverse genetic pool equips banana plants with the resilience needed to navigate through challenges posed by biotic and abiotic factors. In contrast, a lack of genetic diversity renders banana crops vulnerable to extinction, especially in the face of rapidly changing environmental conditions. Within individual genomic groups, banana exhibit varying degrees of genetic diversity, shaped by mutations and decades of selective breeding efforts aimed at enhancing desirable traits (Thierry, 2020). Diversity in plant genetic resources allows plant breeders to develop new and improved banana cultivars with desirable characteristics, including farmer-preferred traits and disease-resistance high-yielding varieties (Govindaraj et al., 2015).

Despite the inherent challenges in preserving genetic diversity within genomic groups, it remains a crucial endeavor for the long-term sustainability of banana cultivation. The conservation of diverse genetic resources serves as the foundation for breeding programs aimed at developing new banana cultivars with enhanced traits, including resistance to diseases and pests, improved yield, and better adaptation to changing environmental conditions.

The domestication of banana spans over a millennium, resulting in the emergence of numerous parthenocarpic varieties. These varieties, characterized by the absence of seeds and developed through natural hybridization, have been propagated vegetatively by farmers over generations. However, it's noteworthy that the initial domestication process likely tapped into only a fraction of the available genetic diversity present in wild banana species (De Langhe et al., 2009). Understanding the genetic diversity of *Musa* species is not only crucial for the preservation of biodiversity but also for addressing future food security challenges (Ortiz, 1997). The advent of CRISPR technology represents a significant leap forward in genetic manipulation, offering unprecedented precision and speed in breeding efforts. This revolutionary tool has the potential to expedite the breeding cycles of banana and facilitate the development of cultivars with tailored traits, thereby contributing to the resilience and sustainability of banana cultivation in the face of evolving environmental and agricultural landscapes.

## 4 Overview of CRISPR/Cas gene-editing technology

The field of gene editing, encompassing technologies that enable precise alterations to an organism’s DNA, has witnessed significant advancements. These tools empower scientists to add, remove, or modify genetic material at specific genomic locations with unparalleled accuracy. Among the various gene editing tools, clustered regularly interspaced short palindromic repeats (CRISPR) and associated protein (Cas) systems have emerged as a cornerstone of genetic manipulation due to their speed, cost-effectiveness, precision, and efficiency, surpassing previous techniques like meganucleases, zinc finger nucleases (ZFNs), and transcription activator-like effector nucleases (TALENs) (Tripathi et al., 2022; Ntui et al., 2023).

CRISPR/Cas9, derived from a naturally occurring bacterial immune defense mechanism against viruses, functions by leveraging RNA segments generated from CRISPR arrays to identify and bind to specific DNA sequences (Koonin and Makarova, 2009). Upon binding, the Cas9 enzyme cleaves the DNA at precise locations, initiating the process of editing. Scientists have harnessed this bacterial defense system to edit DNA by designing short guide RNA (gRNA) sequences that guide the Cas9 enzyme to target DNA sequences in cells. Once the desired DNA sequence is identified, Cas9 cuts the DNA, allowing for the deletion, addition, or substitution of nucleotides, thereby altering the genomic DNA of cells.

The CRISPR/Cas9 system encompasses two classes (Class 1 and Class 2), six types (I to VI), and several subtypes, each with distinct characteristics (Xu and Li, 2020). The CRISPR/Cas9 technology primarily comprises two essential components: the Cas9 nuclease and the gRNA. The gRNA guides the Cas9 enzyme to induce precise double-stranded breaks (DSB) at target sites in the DNA. Moreover, it detects the protospacer adjacent motif (PAM), a three nucleotides sequence, and initiates editing upstream. Subsequently, the cell’s endogenous repair mechanisms, namely non-homologous end joining (NHEJ) and homology-directed repair (HDR), come into play to repair the DNA damage. The NHEJ pathway, an error-prone mechanism, leads to random insertions or deletions (indels) at the cleavage sites, resulting in frameshift mutations and targeted gene knockouts. On the other hand, the HDR pathway enables precise genomic alterations, such as gene knock-in, gene replacement, or insertion of foreign genes or DNA sequences, by employing a homologous DNA repair template. Furthermore, the type of repair determines the classification of editing into three categories: SDN1, SDN2, or SDN3 (Modrzejewski et al., 2019). SDN1 involves random mutations in the host genome, altering gene function or causing gene silencing or knockout. SDN2 utilizes a repair template matching the DSB, leading to nucleotide substitution or targeted indels via HDR. SDN3 facilitates the targeted insertion of foreign genes by repairing the DSB with a longer repair template than the homologous sequences.

## 5 Advances in CRISPR technology

**FIGURE 1:**
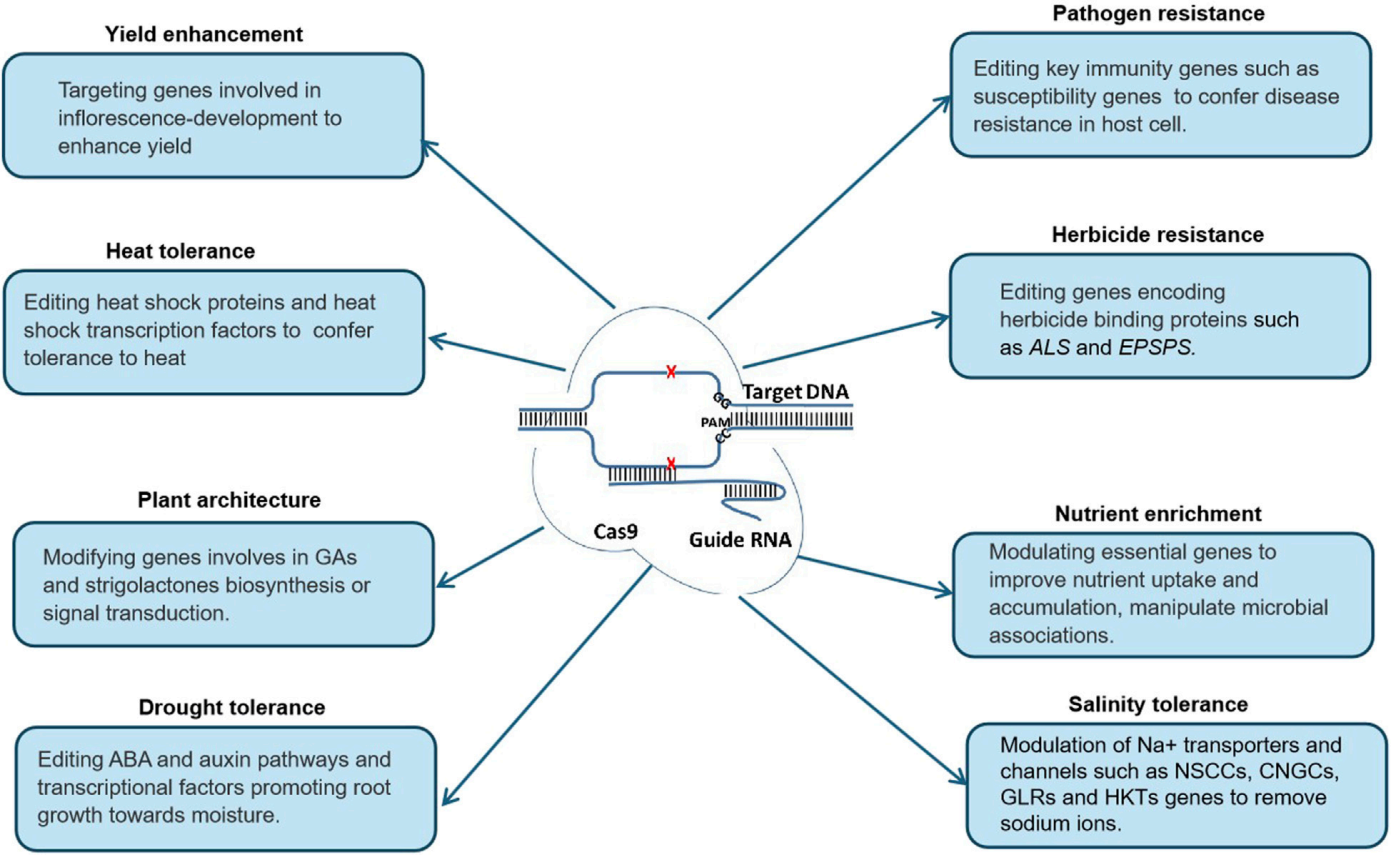
Application of CRISPR/Cas9 based editing in crop improvement.

Since the advent of CRISPR technology, several CRISPR-based tools have been developed, with broadened targeting ranges, enhanced editing specificity and efficiency, and other unique functionalities, revolutionizing crop engineering (Figure 1). CRISPR/Cas9 remains the most widely utilized system due to its stability, adaptability, ease of design, and capacity to multiplex gene editing Originating from the type II CRISPR immune system in bacteria, CRISPR/Cas9 comprises the Cas9 endonuclease from *Streptococcus pyogenes* and a synthetic single guide RNA (sgRNA). The sgRNA directs Cas9 to a specific DNA sequence, guided by the protospacer adjacent motif (PAM), with Cas9 demonstrating a higher affinity for NGG compared to NAG. This system enables precise gene editing and simultaneous modification of multiple genes (Tripathi et al., 2023).

In addition to Cas9, several other Cas variants, including Cas12a, Cas13, Cas14 and CasX have been developed. Cas12a, also known as Cpf1, is a type V, class 2 CRISPR that only harbors the RuvC domain. It possesses RNase activity for crRNA synthesis and DNase activity for single strands. Cas12a recognition of PAM sequences like TTN/TTTN/TTTV and T-rich motifs allows for efficient multiplex gene modifications using a single sequence array on the selected gRNA (Tripathi et al., 2022). Cas13a, a class 2 type VI-A ribonuclease, targets and cleaves single-stranded RNA, offering higher accuracy in viral detection compared to traditional methods. PAM fragments are not necessary for Cas13a activity.

Cas14, an RNA-guided nuclease, uniquely recognizes DNA without PAM dependency, displaying versatile ssDNA cleavage capabilities and high specificity for single-nucleotide polymorphisms (SNPs) (Wu et al., 2022; Zhou et al., 2023). In contrast to Cas12a, Cas14 recognises SNPs with great specificity and sensitivity, which has been used for pathogen discrimination and genotyping. This characteristic has been harnessed for pathogen discrimination and environmental monitoring applications. Indirect sensing of non-nucleic acid targets using Cas14 has been investigated, allowing for the sensitive detection of antibiotics with low nanomolar sensitivity. LC-MS and the usage of metal isotopes, however, made it less straightforward. Wu et al. (2022) created a CRISPR/Cas14-based aptasensor that achieved excellent sensitivity in environmental monitoring by detecting microcystin-LR with nanomaterials-assisted fluorescence generation. However, the use of complementary DNA to block the aptamer’s binding capacity may result in signal loss in target recognition. Furthermore, the potential of CRISPR/Cas14 for aptasensing has never been investigated. Thus, it is worthwhile to try to create an affordable, quick, and direct Cas14-based biosensor for flexible aptasensing.

CasX, identified through metagenomic analysis of groundwater-derived bacteria, represents another promising variant. It functions as an RNA-guided DNA nuclease with a distinct PAM recognition pattern (5′-TTCN) (Liu et al., 2019). CasX is smaller than Cas12, with a unique structure that includes a RuvC domain. Its features include PAM-independent ssDNA trans-cleavage activity, making it a versatile tool for genetic modification. Studies have shown CasX’s potential in gene editing across different organisms, including human cells (Yang and Patel, 2019).

Recently, a new family of RNA-guided endonucleases was found that shares a core domain with the CRISPR/Cas12 family. Evolutionarily conserved, RNA-guided DNA endonucleases carry out a variety of functions *in vivo*, ranging from the prokaryotic TnpB effector to the eukaryotic Fanzor effector. The transposable elements TnpB and Fanzor effectors, together referred to as the OMEGA system, include a CRISPR/Cas9 like domain (RuvC) that functions as an RNA-guided endonuclease (Karvelis et al., 2021). Using uRNA that is complementary to the target DNA, TnpB helps the TnpA module to facilitate the transposition of a particular locus. This RNA guidance allows for the reprogramming of DNA targeting, which is widely employed in gene editing. It is believed that the CRISPR/Cas12 system present in prokaryotes arose from TnpB by introducing extra domains, given that these TnpBs only have a minimal core domain that performs the CRISPR/Cas12 family’s function. From the first classified ISDra2 TnpB, K, and racemifer TnpB types to the most recent database-screened ISDge10, ISAam1, and ISYmu1, the features of target DNA recognition of TnpB have been reported (Altae-Tran et al., 2021; Sasnauskas et al., 2023; Xiang et al., 2023; Badon et al., 2024). Fanzor effectors are mostly found in plants, fungi, protists, arthropods, and eukaryotic viruses. At the molecular level, they exhibit a great deal of similarities to the TnpB system. Fanzor is mainly divided into Fanzor 1 and 2 types. It has been observed that both forms use TAM sequence recognition and uRNA complementary to target DNA, much like TnpB, to produce RNA-DNA heteroduplexes on target DNA. Like TnpB and Fanzor endonucleases, IscB recognises TAM and has a small size of 496 aa (OgeuIscB). Nonetheless, it has comparable functionality, nucleic acid binding, and domain organisation (RuvC, BH, and HNH domains) (Badon et al., 2024).

Prime and base editing technologies represent significant advancements in CRISPR-based gene editing. These editing methods make use of dead Cas9 (dCas9), a modified version of Cas9. To produce a base editor that allows base substitution at single nucleotide resolution without the need for a DNA donor template, a DNA deaminase is coupled to the dCas9 in base editing. The effectors permit C:G-to-T:A or A:T-to-G:C substitution, depending on the kind of DNA deaminase, and the RNA-guided CRISPR system locates the targeted locus in the genome that has to be altered. Prime editing mediates DNA base pair swaps, minor insertions, and tiny deletions (indels) by a process similar to that of classical CRISPR/Cas systems (Matsoukas, 2020; Chen et al., 2021). In contrast, primer editing doesn’t require a donor template or result in DSB; instead, it removes off-target effects and fixes frameshifts brought on by indels. The genome can only be altered by a fusion protein made up of a longer-than-usual gRNA called pegRNA and Cas9 H840A nickase linked to a modified reverse transcriptase (RT) enzyme. Prime and base editing are SDN1 types of editing because they don’t need a DNA donor template. This suggests that they may be treated similarly to non-transgenic crops and may not be subject to stringent biosafety regulations.

These advanced CRISPR technologies offer tremendous potential for improving crop resilience, enhancing nutritional quality, and addressing global food security challenges.

## 6 CRISPR/Cas applications for improvement of banana

Banana is one of the most consumed fruits globally playing a crucial role in food security. However, challenges such as pests, diseases, and nutritional deficiencies pose significant threats to banana production and sustainability. To address these challenges, researchers worldwide are harnessing the power of CRISPR/Cas technology to develop resilient, high-yielding, and nutrient-enriched banana varieties (Table 1).

**TABLE 1:** Summary of gene editing in banana.

S. No.	Trait	Editing system	Target	References
1.	*_*	CRISPR/Cas9	*Musa phytoene desaturase (MusaPDS)*	Kaur et al. (2020), Naim et al. (2018), Ntui et al. (2020)
2.	Xanthomonas wilt resistance	CRISPR/Cas9	*Musa downy mildew resistance 6 (MusaDMR6)*	Tripathi et al. (2021)
3.	Xanthomonas wilt resistance	CRISPR/Cas9	*Musa early nodulin-like 3 (MusaENOD*3)	Ntui et al. (2023)
4.	Banana streak virus resistance	CRISPR/Cas9	*Endogenous Banana Streak Virus* in the B genome of banana	Tripathi et al. (2019c)
5.	Shorter height	CRISPR/Cas9	*gibberellin 20ox2* (*MaGA20ox2*)	Shao et al. (2020)
6.	Increase β-carotene	CRISPR/Cas9	*Musa lycopene epsilon-cyclase* (*LCYε*)	Kaur et al. (2020)
7.	Delayed ripening	CRISPR/Cas9	*Musa acuminata aminocyclopropane-1-carboxylase oxidase (MaACO1)*	Hu et al. (2021)
8.	*—*	Cas-CLOVER	*MusaPDS*	Tripathi et al. (2023)

### 6.1 Targeted gene editing for disease resistance

Researchers at the International Institute of Tropical Agriculture (IITA) in Kenya have spearheaded efforts to enhance banana and plantain varieties through gene editing techniques. By targeting disease susceptibility “S”genes in banana, they have generated hundreds of gene-edited events, many of which have exhibited enhanced resistance to bacterial diseases after rigorous screening in controlled environments (Tripathi et al., 2021; Ntui et al., 2023). These promising events are now slated for field trials before eventual deployment to farmers’ fields.

The availability of reference genome sequences and sophisticated CRISPR/Cas9 gene editing tools has greatly facilitated the development of banana resistant to BSV and BXW (Tripathi et al., 2022). By meticulously targeting endogenous genes, researchers have achieved significant success in conferring resistance to these devastating diseases.

For instance, BXW resistance has been effectively demonstrated in banana through the precise knockout of S-genes. These host genes play a crucial role in enabling pathogen invasion, thereby facilitating pathogen proliferation and symptom emergence. Editing these pivotal S-genes has unlocked broad-spectrum resistance against bacterial pathogens, providing a promising avenue for disease management (Zaidi et al., 2020). Editing S-genes can confer broad-spectrum resistance in certain scenarios and resistance tailored to the specific pathogen (Peng et al., 2017; Kim et al., 2018). Several disease susceptibility genes linked to bacterial resistance have been identified and targeted for editing in banana plants (Tripathi et al., 2020).

In a specific example, *MusaDMR6* gene in the banana cultivar ‘Sukali Ndiizi’ was knocked out at two sites using a multiplexed CRISPR/Cas9 system via *Agrobacterium*-mediated transformation of embryogenic cells. This resulted in *dmr6* mutants displaying increased resistance to BXW without any morphological defects (Tripathi et al., 2021). DMR6 functions as a negative regulator of plant defense, encoding 2-oxoglutarate Fe (II)-dependent oxygenase (2OGO) that hydrolyzes the plant defense signaling molecule salicylic acid (SA), and is upregulated during pathogen infection (Zhang et al., 2017; Low et al., 2020). Loss of function of *DMR*6 gene in other crops, such as tomatoes, has shown to confer resistance to various pathogens (Thomazella et al., 2021).

Similarly, CRISPR/Cas9 was utilized to knockout *early nodulin-like 3* gene (*MusaENOD3*) in the banana cultivar “Gonja Manjaya” to confer resistance to BXW (Ntui et al., 2023). Nodulins and nodulin-like genes are typically induced in legumes upon nodulation by *Rhizobium* bacteria and play a pivotal role in symbiotic interactions. Many nodulin-like proteins, particularly early nodulin-like proteins (ENODL), have been found in various non-leguminous plants, indicating their involvement in processes beyond nodulation, including growth control and nutrient transfer (Denance et al., 2014). Plant defense against infections has been associated with some ENODL proteins. Editing of *MusaENOD3* resulted in edited events exhibiting resistance to BXW. The sequencing data showed several types of mutations, including deletions, insertions, substitutions. Most of the detected deletions were large, ranging from 23 bp to 180 bp (Ntui et al., 2023), underscoring its significance in plant-pathogen interactions and offering novel opportunities for enhancing resistance to bacterial diseases in crops.

Additionally, apart from *MusaDMR6* and *MusaENDOL*, several potential genes identified through comparative transcriptomic studies comparing RNAseq of BXW-susceptible cultivars with BXW-resistance wild progenitor could be targeted for editing using CRISPR/Cas9 to develop resistance against BXW (Tripathi et al., 2019a).

BSV, a member of the badnavirus family (Harper et al., 1999), integrates into the host plant’s genome. It exhibits two forms: the integrated BSV, known as endogenous BSV (eBSV), and the episomal form,. Stress factors like temperature, drought, crossing, and micropropagation cause the integrated viral sequences to become activated, resulting in the infectious episomal form of BSV, which causes symptoms in plants. Drought and extremely high temperatures are two aspects of climate change that might exacerbate BSV disease. A multiplex CRISPR/Cas9 system was employed to inactivate the integrated eBSV by targeting all three open reading frames (ORF) of the virus in “Gonja Manjaya” (Tripathi et al., 2019). The regenerated gene edited events displayed mutations in the target regions that hindered the transcription of viral protein into functional viral episomal proteins. Under water stress, most of the edited events remained asymptomatic compared to the non-edited control plants, demonstrating inactivation of integrated eBSV into infectious viral episomal proteins (Tripathi et al., 2019).

BBTV is a single-stranded DNA (ssDNA) virus with a multipartite genome comprising six circular components with an approximate size of 1.1 kb each (Tripathi et al., 2021). As of now, no instances of CRISPR/Cas-mediated resistance against BBTV have been documented. However, various genes have been edited for resistance against ssDNA viruses, presenting potential targets for developing resistance against BBTV. Here, we describe some promising potential targets for exploration in the quest to establish resistance against BBTV.

Targeting viral proteins has shown promise for tackling DNA viruses. For instance, CRISPR/Cas9 targeting the viral replication-associated region or intergenic region (IR) of *cotton leaf curl Multan virus* (CLCuMuV) and *bean yellow dwarf virus* (BeYDV) have demonstrated effective DNA interference, providing resistance against *beetroot severe curly top virus* (BSCTV) in transgenic *Nicotiana benthamiana* or *Arabidopsis thaliana* plants in bioassay experiments. These mutant viruses were unable to synthesis viral coat proteins and rendered them inactive (Baltes et al., 2015; Ji et al., 2015; Yin et al., 2019). Gene editing of the coat protein (CP) or replicase (Rep) of *tomato yellow leaf curl virus* (TYLCV) by CRISPR/Cas9 resulted in efficient virus interference, as evidenced by the low accumulation of the TYLCV DNA genome in the transgenic tomato and N. benthamiana plants (Tashkandi et al., 2018). Similarly, Ali et al. (2015) observed that sgRNAs targeting the stem-loop sequence were more effective at interfering with multiple geminiviruses, such as the monopartite geminivirus *Cotton Leaf Curl Kokhran virus* (CLCuKoV), the bipartite geminivirus *Merremia mosaic virus* (MeMV), and various severe and mild strains of TYLCV geminivirus in comparison to sgRNAs targeting the viral CP region and the replication-associated region within IR. Viral movement protein (MP) has proven to be a valuable target for developing resistance to viruses. For example, a multiplexed CRISPR/Cas9 system with sgRNAs targeting MP or CP region established resistance to *wheat dwarf virus* (WDV) (Kis et al., 2019).

TR4 is one of the major fungal diseases of banana, which scientists are still battling to control. Gene editing could be a valuable tool for controlling this disease by targeting susceptibility genes. While there is currently no documented evidence of using gene editing to develop resistance to TR4, various susceptibility genes such as *alcohol dehydrogenase* 1 (ADH1), *mildew resistance locus* O (MLO), *LATERAL ORGAN BOUNDARIES* (CsLOB1), *DMR6*, and *ERF922* could serve as potential targets for developing resistance against TR4. These susceptibility genes are induced and overexpressed by pathogens as demonstrated by transcriptome analysis (Tripathi et al., 2019a). Knocking out of these susceptibility genes are proven strategy to generate disease resistant plantls. *ADH1* disruption in tomatoes resulted in reduced ethanol production and impaired growth and development of *F. oxysporum*, indicating its role as a susceptibility gene facilitating pathogen colonization and symptom development, making it a promising target for developing TR4 resistance in banana (Pathuri et al., 2011; Zhang E. et al., 2018).

### 6.2 Improving plant architecture

The plant architecture encompasses the growth and development of a plant from its meristems to the development of stems, leaves, inflorescences and roots. This architecture plays a crucial role in determining their performance and ability to thrive in challenging environments (Wang et al., 2018). One crucial aspect of plant structure is its height, which is influenced by factors like genetic makeup, environmental conditions, and hormone levels such as gibberellins, brassinosteroids, and strigolactones.

Recent breakthroughs in gene editing have provided exciting opportunities to manipulate plant structure for improved outcomes. For example, using CRISPR/Cas9 technology, researchers have successfully targeted genes, GA20ox2, involved in gibberellic acid (GA) signaling in rice, resulting in plants with reduced height by 22.2% and yet yielding 6% more, without affecting other important traits (Han et al., 2019). Similarly, editing the gene CLEAVAGE DIOXYGENASE 7 (CCD7), which controls a crucial step in strigolactone biosynthesis, by CRISPR/Cas9, produced mutants with reduced height and a striking increase in tillers, demonstrating the potential of genetic editing to shape plant morphology (Butt et al., 2018).

In banana cultivation, researchers utilized the CRISPR/Cas9 system to target *MaGA20ox2* gene in the “Gros Michel” variety. This resulted in the development of semi-dwarf mutants with thicker, darker, and greener leaves compared to non-edited plants, highlighting the effectiveness of gene editing in altering plant structure for desired characteristics (Shao et al., 2020).

### 6.3 Targeting genes related to nutrient biosynthesis

A fundamental objective of gene editing in agriculture is enhancing the nutritional content of crops.

Nutritional improvement in cultivated crops is one of the significant goals of gene editing. This can be achieved by augmenting the expression levels of genes involved in nutrient biosynthesis. Editing targets situated upstream of the coding sequences (CDS) or in untranslated regions, like the 5′UTR, which regulate expression, can induce frameshifts leading to premature termination codons, ultimately modulating nutrient production (Nagamine and Ezura, 2022). Furthermore, manipulating metabolic pathway enzymes through gene editing can boost nutrient functionality or aid in metabolizing toxic substances, thereby improving overall crop quality (Nagamine and Ezura, 2022).

Various strategies have been employed to enhance diverse nutrients in crops, including carotenoids, γ-aminobutyric acid (GABA), iron, and zinc. Carotenoids, renowned for their antioxidant properties and role in preventing eye-related diseases, have been a major focus. Beta-carotene, a primary dietary precursor of vitamin A, crucial for eye health and immunity, has been targeted for enhancement using CRISPR/Cas9 in rice, tomato, and banana (Dong et al., 2020; Liu et al., 2021).

Increasing carotenoid levels involves overexpressing *phytoene synthase* genes, like *CrtI* and *PSY*, to redirect carbon flux into the biosynthetic pathway. Conversely, silencing genes such as *LCYe, BCH, ZEP,* and *CCD4* can inhibit precursor conversion. For instance, in banana, Kaur et al. (2020) utilized CRISPR/Cas9 to enhance β-carotene content in “Grand Naine” cultivar by editing the *lycopene epsilon-cyclase* (*LCYε*) gene, resulting in a substantial 6-fold increase (∼24 μg/g) in β-carotene accumulation in the fruit pulp compared to the non-edited plants.

Another area of interest is GABA, an inhibitory neurotransmitter with potential health benefits. Gene editing has enabled the development of GABA-rich foods like the “Sicilian Rouge High GABA” tomato, which accumulates four to five times more GABA than ordinary tomatoes, achieved through targeted deletion of the C-terminal of glutamate decarboxylase (GAD) (Nonaka et al., 2017). Additionally, biofortification of micronutrients like iron and selenium has been demonstrated through gene editing. Targeting genes such as *Vacuolar Iron Transporter* (VIT) and *arsenite tolerant* 1 (*astol1*) in crops like rice has resulted in increased iron and selenium content, respectively, offering potential health benefits to consumers (Che et al., 2021).

### 6.4 Targeting genes related to shelf life

Banana, as a typical climacteric fruit, ripen and decay within a week after exposure to exogenous ethylene. This short shelf life significantly limits their storage, transportation, and marketing, leading to substantial postharvest losses. By editing the *aminocyclopropane-1-carboxylase oxidase* (*MaACO1*) gene, it is possible to delay the ripening process by reducing endogenous ethylene production (Hu et al., 2021). This approach demonstrates significant potential in enhancing banana quality and resilience.

Further, researchers at Tropic Biosciences developed non-browning banana by rendering a key gene responsible for polyphenol oxidase production nonfunctional. This breakthrough holds immense potential to dramatically reduce food waste and carbon dioxide emissions along the supply chain, with projections indicating a potential decrease of over 25%. This is particularly significant given that more than 60% of exported banana currently go to waste before reaching consumers (source: Tropic Biosciences, 2023).

These gene-edited banana have received a non-GMO exemption from the Philippines Department of Agriculture-Bureau of Plant Industry. This marks a significant milestone as the first gene-edited product to undergo the newly established regulatory determination process in the Philippines. As a result of this determination, Tropic’s non-browning banana can now be freely imported and propagated within the country.

## 7 Future prospects for gene editing in banana

### 7.1 Advancements in CRISPR-based gene activation or inhibition

In the context of banana improvement, CRISPR-based gene activation or inhibition holds immense potential for enhancing specific traits. Our research at IITA focuses on activating endogenous banana genes associated with antimicrobial properties, pathogen resistance, and disease tolerance through CRISPRa. By targeting genes identified through transcriptomic analysis (Tripathi et al., 2019a), we aim to confer resistance to diseases like BXW, which poses a significant threat to banana cultivation. Initial screenings of regenerated plants have shown promising levels of gene activation, paving the way for further characterization of their resistance to BXW and other banana diseases.

Moreover, CRISPRi presents a promising strategy for developing virus-resistant banana. When viruses attack plants, they incorporate their genetic material into the genome to reproduce and generate the building blocks for new virus particles. In response, plants activate their RNAi machinery to defend themselves against invading viruses. However, many viruses could inhibit the plant RNAi silencing pathway by releasing a suppressor protein to prevent siRNAs from initiating the defense process (Karlson et al., 2021). By targeting viral RNA, CRISPRi could disrupt viral invasion and enhance plant immunity. Zhang Y.-Z. et al. (2018) produced transgenic Arabidopsis plants resistant to CMV using CRISPRi technology. They showed that the resistance could be detected up to T6 generation. Similarly, Aman et al. (2018) developed a CRISPR/dCas9 construct containing Cas13a, which could innately process pre-crRNA into functional crRNA to target the viral mRNAs and deliver them to tobacco plants. When the plants were inoculated with a recombinant TuMV expressing GFP (TuMV-GFP), they found that the intensity of GFP-expressing TuMV in tobacco was reduced up to 50%, indicating the successful control over the spread of the viral GFP signal (Karlson et al., 2021). Previous studies have demonstrated successful virus resistance in plants like Arabidopsis, and tobacco using CRISPR technology, indicating its potential in banana virus management. With further optimization and refinement, CRISPRi could emerge as a powerful tool for conferring robust viral resistance in banana, safeguarding their production from devastating viral infections.

### 7.2 Alternative CRISPR tool for gene editing in banana

CRISPR/Cas9 has emerged as a pivotal tool for gene editing, holding immense promise for revolutionizing agriculture and addressing critical issues such as climate resilience and food security. However, one of the challenges researchers face is navigating the complexities of intellectual property (IP) protection and licensing to enable the release of gene-edited crops for widespread use by growers. Securing licenses for CRISPR/Cas9 technology can be challenging due to high demand and intricate legal frameworks.

To circumvent these challenges and facilitate the development of gene-edited crops, alternative gene editing approaches with clearer IP issues are being explored. Researchers at IITA have pioneered an alternative gene-editing tool tailored specifically for banana, known as the Cas-CLOVER system (Tripathi et al., 2023). This innovative technology is founded on dual-guide RNA and the programmable clover endonuclease Clo051, which induces double-strand breaks at the target site (Madison et al., 2022).

The Clo051 endonuclease functions as a binding protein at the DNA target site, while the fusion protein comprises an inactivated or dead Cas9 (dCas9) protein. Unlike CRISPR, the Cas-CLOVER system employs two gRNAs along with the Clo051 endonuclease, requiring the dimerization of subunits associated with each gRNA. This dual-guide RNA mechanism ensures highly targeted and precise gene editing, as Clo051 generates double strands only when both gRNAs are simultaneously engaged.

Researchers successfully validated the efficacy of the Cas-CLOVER technology in banana gene editing, particularly targeting mutations in the banana *phytoene desaturase (MusaPDS)* gene (Tripathi et al., 2023). Banana mutants generated through this technique exhibited an albino phenotype, indicative of disrupted *PDS* gene function. This demonstration underscores the precision and versatility of the Cas-CLOVER system for precise gene editing in banana, offering a promising alternative to conventional CRISPR/Cas9 technology.

### 7.3 Potential applications of base editing and prime editing in banana

Base editing and prime editing offer transformative potential for banana improvement, though their application in this crop is still emerging. Base editing, a technique that enables precise nucleotide substitutions, could significantly enhance banana traits, particularly in areas such as nitrogen use efficiency and the production of DNA-free plants. For instance, in rice, a CRISPR/Cas9-xyr5APOBEC1-based system was employed to replace a cytosine with a thymine in the NRT1.1B gene, resulting in improved nitrogen use efficiency (Hu et al., 2015). Similarly, in banana, base editing could be used to enhance nitrogen utilization.

One notable application of base editing is the development of herbicide-resistant, transgene-free plants. A*cetolactate synthase* (*ALS*) gene modulates herbicide resistance in plants. In watermelon, single-base substitutions in *ALS* gene enabled the production of herbicide-resistant, transgene-free plants (Tian et al., 2018). A similar approach could be applied to bananas, where base editing could simultaneously edit the ALS gene and other genes of interest, facilitating the generation of transgene-free plants resistant to herbicides while enabling trait enhancement through multiplexing (Zhang et al., 2019).

Prime editing, which allows precise insertions, deletions, and base substitutions without requiring double-strand breaks or donor DNA, has also demonstrated significant potential in crop breeding. For instance, Jiang et al. (2023) utilized the PPEmax system to generate TAP-IVS mutant rice plants with glyphosate resistance, and Qiao et al. (2023) applied a similar strategy to maize. In addition, efficient insertion of protein tags has been achieved using optimized PE techniques (Li et al., 2023), which could be beneficial for functional genomics in banana.

Prime editing has also shown promise in regulating protein expression through the manipulation of upstream open reading frames (uORFs) in eukaryotes (Zhang et al., 2020). Xue et al. (2023) developed methods to fine-tune uORF expression, which could be applied to banana to precisely regulate target gene expression.

Moreover, prime editing has been successfully employed to confer disease resistance in plants. For example, Gupta et al. (2023) engineered resistance to bacterial blight in rice using enhanced PPE systems, PE5max. Techniques such as knocking in resistance elements or generating resistance alleles could be adapted to develop resistance to BXW, a major disease affecting bananas.

Both base editing and prime editing hold significant promise for advancing banana improvement by enhancing traits such as nutrient efficiency, disease resistance, and enabling the production of transgene-free plants. These innovative editing techniques could address critical challenges in banana cultivation and improve overall crop resilience and productivity.

## 8 Regulatory challenges regarding the commercialization of gene-edited banana and strategy to develop transgene-free banana

The pursuit of transgene-free gene editing in banana aims to create non-GMO plants with desired traits while addressing regulatory constraints associated with GMOs. Despite the broader array of transformation approaches available, achieving transgene-free plants, especially in clonally propagated crops like banana, remains a challenge.

Currently, gene editing in banana involves plasmid delivery, where plasmids containing the Cas9 protein, selection marker genes, promoters, and terminators are introduced into plant cells via *Agrobacterium-*mediated transformation. These gene sequences integrate into the banana genome. Given that banana is vegetatively propagated crop, segregating out these sequences through crossing is impractical. Consequently, gene-edited banana is classified as GMOs by regulatory bodies and are subject to stringent biosafety regulations, which can hinder commercialization and acceptance (Tripathi et al., 2019c). To address this regulatory hazel and increase the commercialization of gene edited banana, it is imperative to produce DNA-free products. Several strategies are being explored to produce transgene-free gene-edited banana plants.

One approach involves utilizing ribonucleoproteins (RNPs), where a preassembled complex of Cas9 protein and gRNA is delivered into the plant cell (Liang et al., 2017). This complex facilitates gene editing at target sites immediately after transfection and is rapidly degraded by endogenous proteases, minimizing off-target effects and preventing the integration of foreign DNA elements (Woo et al., 2015). Various delivery methods such as electroporation, particle bombardment, and protoplast transfection have been explored for direct delivery of the RNA-guided engineered nucleases- ribonucleoproteins (RGENs-RNPs) into plant cells, with protoplast transfection being the most versatile. While some authors have reported the regeneration of complete plants from banana protoplast (Panis et al., 1993; Matsumoto and Oks, 1998; Assani et al., 2001), regenerating plants from banana protoplasts remains challenging (Tripathi et al., 2022).

Another strategy involves transiently delivering the editing machinery into plant cells via *Agrobacterium* without applying selection. This method has been demonstrated in other crops, resulting in the production of transgene-free plants. For example, Chen et al. (2018) produced transgene-free tobacco plants by transient expression of CRISPR/Cas9 containing gRNAs targeting the PDS gene. They obtained up to 8.2% non-transgenic mutants. Using a similar approach, Veillet et al. (2019) modified the *acetolactate synthase* (ALS) gene via a cytidine-based editor and obtained transgene-free potato and tomato plants with mutation efficiency of 10% and 12.9%, respectively. However, challenges such as high off-target effects and the need for extensive screening remain.

To address these challenges, researchers are designing plasmids with mechanisms for T-DNA excision and removal following editing (Dalla Costa et al., 2020). Techniques such as the Flp/FRT system and synthetic cleavage target sites (CTS) have been developed to remove T-DNA from CRISPR-edited plants. Although challenges with trimming at T-DNA boundaries exist, these approaches represent significant progress toward producing transgene-free plants.

In ongoing research, efforts are underway to refine the process of producing transgene-free banana through transient delivery of the Cas9-gRNA reagent by *Agrobacterium*. Additionally, procedures for regeneration, PEG transfection, and protoplast isolation are being developed to streamline the process.

While transferring CRISPR/Cas9 plasmids into germ lines or protoplasts presents technical challenges and inefficiencies, innovative approaches such as *de novo* induction of meristems offer promising avenues for overcoming these limitations in dicotyledonous plants. This approach involves delivering developmental regulators and gene-editing components into somatic cells of entire plants, resulting in the transmission of desired DNA modifications to the next-generation. The graft-mobile gene editing system can be another strategy to the production of transgene-free plants in one generation without the need for transgene segregation (Yang et al., 2023).

## 9 Ethical issues of gene editing in agriculture

Gene editing is predicted to usher in a new Green Revolution, enhancing food and nutritional security worldwide and mitigating the effects of climate change. However, the technology also raises significant ethical concerns that encompass environmental, social, and economic dimensions.

One major concern is the possibility of off-target effects, edits occurring in unintended locations, which can result in unwanted phenotypes. The aspect of safety has been one of the critical issues of gene editing. There are questioned surrounding the safety of gene editing, especially when the technology is used for gene drives, since off-target effects in gene editing are not fully understood. There is also concern that gene-edited crops could reduce biodiversity if they outcompete natural species or if large-scale monoculture practices are adopted.

Another significant issue is the regulatory framework for gene editing. There is ongoing debate over whether gene-edited crops should be categorized as GMOs (Karalis et al., 2020). While some countries have clarified their regulations regarding gene edited crops, others, such as the European Union, South Africa and New Zealand, maintain very strict regulations, leading to variations in national regulatory strategies. Policy and regulation development for gene editing in plant breeding must also consider factors like farmers' rights and public acceptance (Idris et al., 2023). As with many new technologies, there is concern of Intellectual property rights (IPR). Gene-edited products will inevitably be patented, providing owners with IPR rights—typically agri-food corporations—with what amounts to monopolistic control over the gene-editing process' output (Sprink et al., 2022). The patenting of gene-edited crops by corporations can lead to concerns about farmers' rights and their dependency on a few large companies for seeds, potentially driving up costs and limiting traditional farming practices.

Moral and religious objections also play a significant role in the ethical debate. Many people believe that gene editing interferes with natural creation, equating it to “playing God.” These objections highlight the need for inclusive and culturally sensitive discussions when considering the widespread adoption of gene editing in agriculture.

## 10 Conclusion

CRISPR/Cas based gene editing stands as a transformative technology with vast potential for enhancing crop productivity and nutritional quality, thus bolstering global food security amidst mounting environmental challenges. However, the clonal propagation of banana presents unique challenges in integrating gene-edited traits due to the seedless nature of the fruit. While plasmid-based delivery systems and embryogenic cell methods offer feasible pathways for generating gene-edited banana plants, the task of segregating transgenes through conventional breeding is hindered by the lack of seeds in banana. Overcoming these hurdles requires optimization of techniques like using RNPs for generating plants from protoplasts or microprojectile bombardment of cell suspension. Additionally, robust protoplast regeneration systems must be developed through further research to facilitate the creation of transgene-free plants in banana cultivars with shorter breeding cycles. Disease resistance trait is successfully targeted by knocking off the susceptible genes in the banana genome, like DMR6 and Early Nodulin gene. However, challenges persist in targeting complex polygenic traits like abiotic stress tolerance, necessitating the simultaneous knockout of multiple genes or targets.

Nevertheless, the diligent exploration of innovative technologies such as CRISPR/Cas holds promise for delivering high-yielding better banana with enhanced nutritional content and disease resistance. By integrating precision genetics with traditional breeding programs and adopting transgene-free strategies, researchers can unlock the full potential of gene-edited banana. The future holds exciting prospects for the development of banana that not only thrive in diverse environments but also offer superior nutritional value, benefiting farmers and consumers alike.
